# Are Early Warning Scores Useful Predictors for Mortality and Morbidity in Hospitalised Acutely Unwell Older Patients? A Systematic Review

**DOI:** 10.3390/jcm7100309

**Published:** 2018-09-28

**Authors:** Romesh Jayasundera, Mark Neilly, Toby O. Smith, Phyo Kyaw Myint

**Affiliations:** 1 Institute of Applied Health Sciences, University of Aberdeen, Aberdeen AB25 2ZD, Scotland, UK; romesh.jayasundera@nhs.net (R.J.); mark.neilly@nhs.net (M.N.); 2 Nuffield Department of Orthopaedics, Rheumatology and Musculoskeletal Sciences, University of Oxford, Oxford OX3 7LD, UK; toby.smith@ndorms.ox.ac.uk

**Keywords:** EWS, vital signs, geriatrics, older people, mortality, morbidity, critical care

## Abstract

Background: Early warning scores (EWSs) are used to identify deteriorating patients for appropriate interventions. We performed a systematic review to examine the usefulness of EWSs in predicting inpatient mortality and morbidity (transfer to higher-level care and length of hospital stay) in older people admitted to acute medical units with sepsis, acute cardiovascular events, or pneumonia. Methods: A systematic review of published and unpublished databases was conducted. Cochrane′s tool for assessing Risk of Bias in Non-Randomised Studies—of Interventions (ROBINS-I) was used to appraise the evidence. A narrative synthesis was performed due to substantial heterogeneity. RESULTS: Five studies (*n* = 12,057) were eligible from 1033 citations. There was an overall “moderate” risk of bias for all studies. The predictive ability of EWSs regarding mortality was reported in one study (*n* = 274), suggesting EWSs were better at predicting survival, (negative predictive value >90% for all scores). Three studies (*n* = 1819) demonstrated a significant association between increasing modified EWSs (MEWSs) and increased risk of mortality. Hazards ratios for a composite death/intensive care (ICU) admission with MEWSs ≥5 were significant in one study (*p* = 0.003). Two studies (*n* = 1421) demonstrated that a MEWS ≥6 was associated with 21 times higher probability of mortality (95% Confidence Interval (CI): 2.71–170.57) compared with a MEWS ≤1. A MEWS of ≥5 was associated with 22 times higher probability of mortality (95% CI: 10.45–49.16). Conclusion: Increasing EWSs are strongly associated with mortality and ICU admission in older acutely unwell patients. Future research should be targeted at better understanding the usefulness of high and increasing EWSs for specific acute illnesses in older adults.

## 1. Introduction

Early warning scores (EWSs) are composite scales which consider patients’ vital signs such as blood pressure and heart rate. They are often used in the hospital setting to assess worsening or improvement in patients’ clinical status over time. Higher scores are associated with a need for further treatment or escalation to intensive care unit (ICU) or high dependency unit (HDU) care [1,2]. EWSs can be calculated by healthcare assistants or nursing staff, who can contact an appropriate medical team member depending on the patient’s score with the view of treatment escalation as indicated [3,4]. Accordingly, EWSs are used worldwide as a bedside monitoring tool in acute settings [3,5,6,7,8].

EWSs encompass respiratory rate (RR), oxygen saturation (SpO_2_), temperature, blood pressure (BP), and heart rate (HR). Consciousness level is also often assessed and typically uses the alert/responds to voice/pain/unresponsive (AVPU) system [9,10,11]. In the acute medical setting, identification of a deteriorating patient is vital so that timely, clinically effective treatment may be initiated. This is true for a variety of conditions such as sepsis, myocardial infarction (MI), and cerebrovascular events, including stroke. Increasing EWSs indicate deteriorating patients and thus it is also used to select patients who may benefit from a higher level of care, such as intensive care unit (ICU) or high dependency unit (HDU) admission [1].

There has been an increase in the number of acute medical admissions, particular amongst older people [12]. In England alone, 50% of older people seen in Accident and Emergency (A&E) are admitted, compared to 16% of people younger than 65 years [13]. Conditions such as sepsis [14], MI, stroke [15], and pneumonia [16], are leading causes of mortality and morbidity in older people globally. To date, the evidence surrounding the usefulness of EWSs as predictors of mortality and morbidity in older adults admitted with medical illness has not been systematically assessed. Given that EWSs are used worldwide, [6,7,8,17,18], evaluation of the value of these tools is warranted.

The purpose of this systematic review was therefore to determine whether EWSs are accurate tools for predicting in-patient mortality and morbidity in older patients admitted to hospital with sepsis, acute cardiovascular event (MI, stroke), or pneumonia.

## 2. Materials and Methods

The review protocol was registered a priori through the PROSPERO databases (registration number: CRD42016051351).

### 2.1. Search Strategy and Selection Criteria

The databases MEDLINE, AMED, EMBASE, CINAHL, OpenGrey, Clinicaltrials.gov, WHO Trial registry, PubMed, and Web of Science were searched from their date of inception to February 2018. The search strategy for MEDLINE is presented in Appendix A and was modified appropriately for each database.

Observational studies including prospective cohort studies, retrospective cohort studies, and case series were eligible study designs. The population under investigation included persons aged 65 years and older who were admitted to an acute hospital. There was no restriction on gender or whether individuals lived in their own home or care home prior to hospital admission. Patients were required to be admitted with one of the following conditions: sepsis and/or acute cardiovascular event (MI, stroke), and/or pneumonia.

Studies were eligible if they presented data on mortality or morbidity. The primary outcome was mortality reported at various time-points (inpatient, early mortality, or mortality post-discharge). Secondary outcomes included morbidity (including whether a patient was transferred to HDU or ICU) and acute hospital length-of-stay.

### 2.2. Identification of Eligible Studies

Two reviewers (RJ/MN) independently reviewed the titles and abstracts from the search results. The full-texts of all potentially eligible studies were independently reviewed by the same two reviewers against the inclusion/exclusion criteria. Where disagreement arose a third reviewer (TS) acted as an adjudicator.

### 2.3. Data Extraction

Two reviewers (RJ/MN) independently extracted data from all eligible studies and compared results to ensure consistency. Data for extraction were decided a priori and included: population characteristics (age, gender, reason for hospital admission), type and components of the EWSs, whether an EWS comparison was used, and what outcome measures were adopted.

### 2.4. Quality Assessment

Risk of bias was assessed using the Cochrane’s tool for assessing Risk of Bias in Non-randomised Studies—of Interventions (ROBINS-I) [19,20]. This was performed independently by two reviewers (RJ/MN). Where discrepancy arose, a third reviewer (TS) acted as an adjudicator.

### 2.5. Data Analysis

Study heterogeneity was explored by visual inspection of the data extraction tables to assess variability in the recruited populations, study design and EWS used. This identified considerable heterogeneity between the included studies. Accordingly, a narrative analysis was conducted rather than evidence synthesis using meta-analysis techniques. Thus, we analysed the findings in relation to the risk of mortality and morbidity using the EWS criteria as specified in the included papers. Accordingly, odds ratio (OR) and hazard ratio data with 95% confidence intervals (where available) were gathered. Due to the limited number of studies identified, it was not possible to undertake the planned subgroup analyses, which aimed to assess the usefulness of the EWS across cohorts with different ages, indications for hospital admission, and for those who did not die during their hospital admission.

## 3. Results

### 3.1. Search Strategy

**Figure 1:**
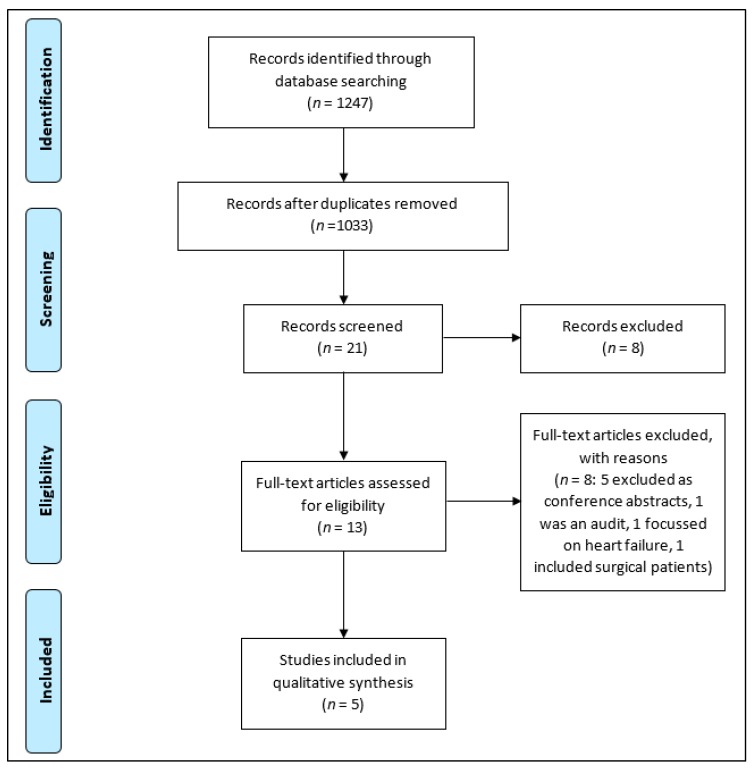
PRISMA flow diagram [23].

The search results are summarised in Figure 1. In total, 1033 studies were identified from the initial search. Of these, 13 studies were deemed potentially eligible. After review of full texts, five studies met the eligibility criteria and were included.

### 3.2. Quality Assessment

The ROBIN-I critical appraisal results are presented in Table 1. From this, there was an overall “moderate” risk of bias rating for all studies. Recurrent strengths in the evidence base are that there was a low risk of bias for reporting bias, deviations to the planned study interventions, and reporting of study methods. There was however moderate risk of measurement bias across all five studies and moderate risk of selection bias in Cei et al. [5]. Whilst Huggan et al. [7] and Alrawi et al. [21] had low risk of bias for confounding, this was judged as moderate risk in Cei et al. [5], as a serious risk in Kellett and Deane [22], and as a critical risk in Liljehult and Christensen [6].

### 3.3. Characteristics of Included Studies and EWS

A summary of the included study characteristics is presented in Table 2.

Liljehult and Christensen [6], and Cei et al. [5] both used admission EWSs and MEWSs, respectively. Liljehult and Christensen [6] also gathered data on maximum EWSs. Liljehult and Christensen [6] calculated sensitivity, specificity, positive and negative predictive values, and area under receiver operating characteristics (AUROCs) for admission, and maximum EWSs (scores ranging from 0 to 10). Furthermore, these authors [6] subdivided scores into low, moderate and high risk (0–1; 2–3; ≥5, respectively), whereas Cei et al. [5] categorised scores as 1, 2, 3, 4, ≥5.

Huggan et al. [7] assessed ICU or HDU admission or death as outcomes against EWS data. They specifically analysed the association between clinical outcome and a MEWS ≥5, abnormal SBP, RR, HR, temperature, and AVPU. Kellett and Deane [22] devised a “simple clinical score”, which included, among others, the variables used for EWSs. They identified independent predictors of mortality using logistic regression and devised a 16-variable clinical score which included RR, SpO_2_, temperature, HR, BP, and altered mental state.

### 3.4. Mortality

All five included studies reported an association between EWSs [6], MEWSs [5,7,21,22], and mortality, as shown by increasing mortality rate with increasing scores. Liljehult and Christensen [6] reported a total of 24 deaths. Mortality rates were lowest for admission EWSs 0–1 (2%) and highest for admission EWS ≥5 (63%). There was no significant difference between AUROC for admission and maximum EWS. Liljehult and Christensen [6] reported that RR and AVPU were significant predictors of mortality in their cohort. Positive predictive values (PPVs) for admission and maximum EWSs also increased with increasing scores. Negative predictive values (NPVs) were >90% at all scores [6].

Fifty-five people in the Alrawi et al. [21] cohort died within seven days of hospital admission. They reported that a MEWS >3 was significantly associated with increased odds of death (adjusted OR: 12.03 at MEWS 4–5; OR: 21.49; MEWS ≥6).

Cei et al. [5] reported 102 deaths and reported that increasing MEWSs were significantly associated with increasing mortality. Both Alrawi et al. [21] and Cei et al. [5] assessed the odds of death using a MEWS. Alrawi et al. [21] categorized MEWSs (up to 6+), whereas Cei et al. [5] analysed scores as a continuous variable (up to 5+). Both studies reported a significantly increased odds of death with increasing MEWSs (MEWSs 6+ = OR: 21.49 [21]; MEWSs ≥5 = OR: 22.59 [5]).

Kellett and Deane [22] reported 316 deaths within their cohort. Whilst they did not investigate EWS, they calculated a non-validated MEWS (one point for altered mental state and no coma; two points for coma) and found an AUROC of 64.7%. For each EWS variable included in their clinical score [22] there was a significant association between mortality and abnormal values, with the exception of HR <40 which was found to be non-significant.

### 3.5. Morbidity

Two studies investigated the usefulness of MEWSs on morbidity within this population [5,7]. Huggan et al. [7] assessed the value of MEWSs on length of stay and reported that abnormal consciousness levels were significantly associated with excess length of stay (defined as length of stay >7 days) (*p* = 0.003).

### 3.6. Composite Mortality/Morbidity Endpoint

Sixteen patients in Huggan et al. [7] were either transferred to HDU/ITU care or died. Based on this small subgroup, Huggan et al. [7] reported that MEWSs ≥5, systolic BP ≥1, and RR ≥2 were significantly associated with death/ICU admission. Similar conclusions were also made by Cei et al. [5] with regard to the composite outcome of mortality/transfer to higher-level care with MEWSs.

## 4. Discussion

Our systematic review found that increasing EWS/MEWSs are significantly associated with inpatient mortality in older patients [5,6,7,21,22]. There is an association between EWSs/MEWSs and transfer to higher-level care (ICU) [5,7]. There is limited evidence on the predictive ability of these scores on this outcome. The evidence base has a moderate risk of bias and therefore these results should be viewed with caution.

Alrawi et al. [21] reported an association between increasing MEWSs and mortality but with wide confidence intervals, possibly reflecting the limited cohort size. However, both Alrawi et al. [21] and Cei et al. [5] showed increased odds of mortality with increasing MEWSs. Furthermore, both studies assessed the mortality outcome for similar time points (around seven days) post admission. Therefore, their findings are fairly comparable and offer some evidence on the association of increasing MEWSs and increased risk of mortality.

In the Liljehult and Christensen [6] study, EWSs were shown to significantly predict mortality. Nonetheless, there was evidence of a high risk of false positive results with increasing EWSs. Due to NPV >90% for all scores, their results suggest that apart from an EWS of >6 on admission or an EWS of 10, the EWS is better at predicting survival compared to predicting mortality in older patients with an acute stroke. Although assessed in a different population using different statistical approaches, Cei et al. [5] also reported a significant association between increasing MEWSs and increased risk of mortality compared to MEWSs of 0. This strong association with increasing MEWSs and mortality hints at the predictive usefulness of MEWSs.

It has been proposed that EWSs are particularly valuable as they allow members of the multidisciplinary team, principally nursing staff, to quantify a patient’s clinical deterioration and empower escalation of medical care [24]. Whilst this escalation may facilitate early treatment, it may further encourage medical staff to consider earlier HDU or ITU admission [2]. This is important given that a prolonged stay on general wards, prior to ICU admission, is reportedly associated with higher risk of death [25]. Hence, a reliable clinical scoring system that allows for early detection, evaluation and treatment/escalation of acutely deteriorating patients may be advantageous [26]. It is also important to consider that increasing EWSs may facilitate more timely adoption of palliative treatments where indicated [21,22].

This study has one significant limitation. We initially planned subgroup analyses based on age and admitting medical condition. The aim of this was to better understand when and in what context EWS may have greater predictive capability on mortality and morbidity. Due to a paucity of literature, it was not possible to undertake such analyses. As the evidence base develops, it is anticipated that this current limitation may be overcome such that the potential value of EWSs can be better understood for acute medical care.

Whilst not study limitations, two factors arising from the included studies limited the strength of this review’s recommendations. Firstly, the heterogeneity of evidence precluded a direct statistical comparison of the included studies using a meta-analysis. With the exception of Kellett and Deane [22], the majority of patients in the studies are noted as older people; however, no studies could be sourced which completely satisfied our criteria. Finally, SpO_2_, which is included in the standard EWS in the United Kingdom, [9] was not included in the EWS used Huggan et al. [7] or Cei et al. [5], thereby reducing the generalisability of these findings to U.K. practice.

## 5. Conclusions

Although the results from this review do not offer clarity on the predictive ability of EWSs on mortality and morbidity, they do provide assurance of an association between increasing EWSs and mortality and morbidity. Future research should focus on the weaknesses of the current evidence base identified in this review, including understanding the relationship between EWSs and outcomes from specific conditions which have varying prognosis in older people (e.g., pneumonia versus stroke), and to address the lack of trial evidence of management strategies to be used for those with increasing EWSs. This study, therefore, provides an incentive to clinicians and researchers to better understand the usefulness of EWSs and how targeted intervention strategies may be tested in future clinical trials to improve clinical outcomes for older people.

## Figures and Tables

**Table 1:** Summary of quality assessment using the Risk of Bias in Non-Randomised Studies—of Interventions (ROBINS-I) tool.

	Pre-Intervention		At Intervention	Post-Intervention				
Study	Bias Due to Confounding	Bias in Selection of Participants	Bias in Classification of Interventions	Bias Due to Deviations from Intended Interventions	Bias Due to Missing Data	Bias in Measurement of Outcomes	Bias in Selection of Reported Result	Bias Across Domains
**Liljehult and Christensen [6]**	Critical	Low	Low	Low	Moderate	Moderate	Low	Moderate
**Huggan et al. [7]**	Low	Low	Low	Low	Low	Moderate	Low	Moderate
**Alrawi et al. [21]**	Low	Low	Low	Low	Low	Moderate	Low	Moderate
**Cei et al. [5]**	Moderate	Moderate	Low	Low	Low	Moderate	Low	Moderate
**Kellett and Deane [22]**	Serious	Low	Low	Low	Low	Moderate	Low	Moderate

**Table 2:** Summary of study characteristics. EWS: early warning score.

Study	Population	Intervention	Outcomes	Total Number of Patients (*n*)	Age in Years	Country	Gender
**Liljehult and Christensen [6]**	Acute stroke admissions	EWS	Mortality within 30 days	274	72.3 (12.7)	Denmark	50% female
**Huggan et al. [7]**	Admissions to the acute medical ward	Modified EWS (MEWS)	(1) Death/higher level care admission (composite) (2) Excessive length of hospital stay (>7days)	398	64.2 (10.2)	Singapore	52% female
**Alrawi et al. [21]**	Admission to the acute medical unit from nursing homes	MEWS	Deaths within first week of admission.	314	84.2 (8.3)	United Kingdom	68% female
**Cei et al. [5]**	Admissions to medical ward from the emergency room or emergency medicine	MEWS	(1) In-hospital mortality (2) Mortality/ transfer to higher level care (composite)	1107	89.9% >64 years	Italy	56.1% female
**Kellett and Deane [22]**	Acute admissions to the medical unit	Simple clinical score	Mortality within 30 days	Derivation cohort = 6736 (Validation = 3228)	61.9 (20.3)	Ireland	47.4% female

## Appendix A

1. Early warning score.ti.ab 2. Vital sign.ti.ab 3. Physiolog$ score.ti.ab 4. Bedside measure$.tia.ab 5. National early warning score.tia.ab 6. Modified early warning score.ti.ab 7. Scottish early warning score.ti.ab 8. Physiological warning score.ti.ab 9. OR/1-8 10. Mortal$.ti.ab 11. Morbid$.ti.ab 12. Complicat$.ti.ab 13. Adverse event.ti.ab 14. Length of stay.ti.ab 15. Ward transfer.ti.ab 16. High-dependency.ti.ab 17. Critical care.ti.ab 18. OR/10-17 19. In-patient.ti.ab 20. Inpatient.ti.ab 21. Hospital.ti.ab 22. Acute.ti.ab 23. OR/19-22 24. AND/9,18,23 25. Deduplicate

The MEDLINE search strategy which was modified for each of the adopted database searches.
